# Quality of Reporting and Adherence to ARRIVE Guidelines in Animal Studies for Chagas Disease Preclinical Drug Research: A Systematic Review

**DOI:** 10.1371/journal.pntd.0004194

**Published:** 2015-11-20

**Authors:** Julián Ernesto Nicolás Gulin, Daniela Marisa Rocco, Facundo García-Bournissen

**Affiliations:** 1 Servicio de Parasitología y Enfermedad de Chagas, Hospital de Niños Dr. Ricardo Gutiérrez, Buenos Aires, Argentina; 2 Consejo Nacional de Investigaciones Científicas y Técnicas (CONICET), Buenos Aires, Argentina; 3 Agencia Nacional de Promoción Científica y Tecnológica, Ministerio de Ciencia, Tecnología e Innovación Productiva, Buenos Aires, Argentina; Harvard School of Public Health, UNITED STATES

## Abstract

Publication of accurate and detailed descriptions of methods in research articles involving animals is essential for health scientists to accurately interpret published data, evaluate results and replicate findings. Inadequate reporting of key aspects of experimental design may reduce the impact of studies and could act as a barrier to translation of research findings. Reporting of animal use must be as comprehensive as possible in order to take advantage of every study and every animal used. Animal models are essential to understanding and assessing new chemotherapy candidates for Chagas disease pathology, a widespread parasitic disease with few treatment options currently available. A systematic review was carried out to compare ARRIVE guidelines recommendations with information provided in publications of preclinical studies for new anti-*Trypanosoma cruzi* compounds. A total of 83 publications were reviewed. Before ARRIVE guidelines, 69% of publications failed to report any macroenvironment information, compared to 57% after ARRIVE publication. Similar proportions were observed when evaluating reporting of microenvironmental information (56% vs. 61%). Also, before ARRIVE guidelines publication, only 13% of papers described animal gender, only 18% specified microbiological status and 13% reported randomized treatment assignment, among other essential information missing or incomplete. Unfortunately, publication of ARRIVE guidelines did not seem to enhance reporting quality, compared to papers appeared before ARRIVE publication. Our results suggest that there is a strong need for the scientific community to improve animal use description, animal models employed, transparent reporting and experiment design to facilitate its transfer and application to the affected human population. Full compliance with ARRIVE guidelines, or similar animal research reporting guidelines, would be an excellent start in this direction.

## Author Summary

There is a growing concern about animal use for scientific purposes. In order to maximize impact of results obtained and avoid unnecessary experiment repetition, certain strategies have been implemented such as publication of guidelines to encourage detailed reporting of animal studies. We contrasted information reported in publications assessing new compounds for Chagas disease in animal models with the suggestions contained in the ARRIVE guidelines. A significant lack of compliance with the guidelines was observed even years after ARRIVE publication, including lack of minimally expected information. These results illustrate the importance of promoting inclusion of detailed information on animal use, in Chagas disease research in particular, but also in all research reporting in general.

## Introduction

Chagas disease (also known as American Trypanosomiasis) is a widespread condition, caused by the hemoprotozoa parasite *Trypanosoma cruzi*, affecting approximately 8 million people worldwide[1]. Formerly considered an endemic illness in South America, it recently became recognized as a global public health concern due to migratory movements [2].

Available drugs for Chagas disease, Nifurtimox (NFX) and Benznidazole (BZ), were developed more than 30 years ago. Although their efficacy in the acute phase of the infection is well documented, clinical outcomes in chronic stages are more variables [2], and occurrence of adverse events is common, especially in adults. Therefore, there is a considerable need for new compounds to improve Chagas disease chemotherapy [3].

Animals models have commonly been employed to study mechanisms involved in pathogenesis, immunological response, and to estimate efficacy of new chemotherapies and vaccines for Chagas disease, among others [4].

Variability of animal models for Chagas disease, and the heterogeneity in readout methods used to define drug response (e.g. parasitemia, PCR in blood, PCR in blood and in tissues) have led to highly variable results when evaluating new drug candidates. This wide gap between results in preclinical research and high failure rate in clinical trials may be explained, in part, by the scarce information contained in most experiments that employ laboratory animals, in which crucial information related to species, strains, genetic background, microbiological status, husbandry conditions and procedures are not properly described or even missed in some occasions.

Inaccurate description of materials and methods and failure to report results appropriately has significant scientific, ethical and economic implications both for the research community and the public opinion. Furthermore, detailed reporting of animal use in scientific papers has a direct connection with the “3Rs’ Principles” of humane use of animals in scientific research (i.e. Replacement, Reduction and Refinement) since a complete and systematic description of what was done and what was found in the experiments may avoid unnecessary repetition [5], facilitate systematic revisions before new essays involving animals are carried out,[6] and simplify comparisons and data integration from different studies [7].

The Animals in Research: Reporting *in vivo* Experiments (ARRIVE) Guidelines were published in June 2010. The main objectives of the ARRIVE guidelines are to improve the quality of animal use reporting in scientific publications to maximize the availability and utility of information gained from every animal in every experiment, preventing unnecessary animal use, and to allow an accurate critical review of animal experiments, making results easier to compare among different research groups to validate and contextualize results to promote translational research to patients’ benefit [8].

The ARRIVE Guidelines consist in a checklist describing the minimum information that all scientific publications using animals should include, such as number and specific characteristics of animals employed; details of housing, husbandry and procedures; experimental design, statistical and analytical methods [8].

The main objective of this systematic review was to evaluate the degree of compliance with ARRIVE guidelines of scientific publications assessing efficacy of new chemotherapy for *T*. *cruzi* in animal models. The secondary objective was to compare these results to information presented in similar papers published before the ARRIVE guidelines were made available.

## Materials and Methods

### Publication search strategy

A systematic search was carried out in PubMed database (National Library of Medicine, USA) to identify potentially relevant scientific papers reporting original research on efficacy of new drugs for Chagas disease using animal models.

A modified filter suggested by Hooijmans et al. was used to find all studies in PubMed reporting animal experiments to evaluate drugs for Chagas disease [6]. Modifications consisted in restricting the search only to studies including mammals. The following MeSH (Medical Subject Headings) terms and connectors were used: Chagas disease OR trypanosoma cruzi AND chagas disease/drug therapy AND animal model.

**Fig 1:**
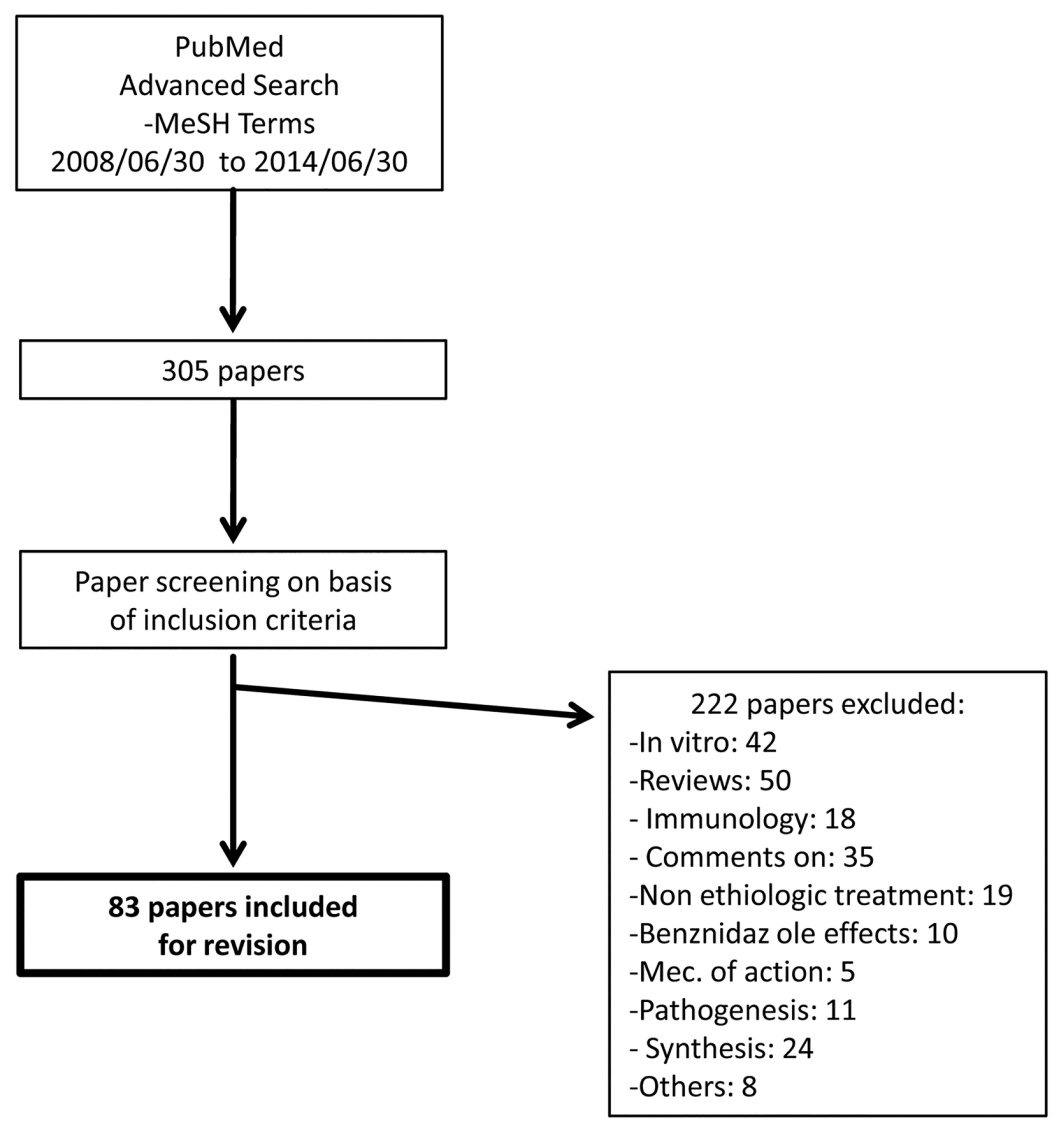
Flow diagram summarizing search strategy.

In order to compare information present in papers published before and after ARRIVE guidelines became widely available the search was performed between 2008/06/30 to 2011/06/30 (i.e before ARRIVE publication) and between 2011/07/01 until 2014/06/30 (i.e. after publication), respectively. The dates for the search period after ARRIVE guidelines publication were set one year after ARRIVE guidelines actual publication to allow scientific community (researchers, reviewers, editors and journals) to adopt them (Fig 1).

Abstracts were reviewed manually and the ones which did not meet inclusion criteria were discarded. Supplementary Material (S2 Fig) illustrates search filters used.

### Evaluation of publications

Relevant publications fulfilling inclusion criteria were randomly assigned to independent reviewers, ensuring that revision was done blindly until the final compilation of results.

The ARRIVE guidelines were used to analyze the papers focusing on the “Material and methods” section to evaluate the degree of compliance of the publicatons with the guidelines. Issues addressing animal model information, husbandry conditions, ethics and strategies implemented to follow 3R’s Principles were compared to the checklist.

Refined approaches established to avoid or minimize pain or stress such as days acclimation before the study starts, refined oral administration with minimum volume and / or oral gavage replacement with a pipette tip instead of oral gavage were considered. Also, anticipated end points determined by parasitaemia peak or severe adverse drug effects were included as refinement strategies.

### Statistics

Reported information rates before and after the ARRIVE guidelines publication were compared using Chi-square test. P values < 0.05 were considered statistically significant in all cases. Statistical calculations were performed in R version 3.1 (The R Foundation for Statistical Computing ISBN 3-900051-07-0).

## Results

A total of 39 articles (out of 176 identified by the search terms) fulfilled inclusion criteria in the period before ARRIVE guidelines publication, and 44 (out of 129 identified) fulfilled inclusion criteria in the period after the guidelines were published.

Supplemental material 1 (S1 Fig) summarizes number of papers included in this review, by publication year. S1 Text contains the complete list of supporting references.

### Information about animals used

Before ARRIVE guidelines publication, animal models for Chagas disease were more diverse. Even though Mouse (*Mus musculus*) was the most popular species used (34 / 39), some papers reported studies on Rat (*Rattus norvegicus*) and Dog (*Canis lupus familiaris*). When Mouse models were used, inbred strains were used slightly more than outbred stocks (18 / 32 vs. 14 / 32).

After ARRIVE guidelines were published, the only animal species reportedly employed to assess *in vivo* efficacy of new compounds for Chagas disease was the Mouse. Half of the publications used inbred strains (22 / 44). There was a considerable predominance of BALB/c strains and Swiss mice stocks in the inbred and outbred experiments, respectively. Table 1 shows in detail all animal models and strains employed.

**Table 1:** Detail of animal models.

	Before-ARRIVE		After-ARRIVE	
Animal species	Ratio	%	Ratio	%
**Mice**	34 / 39	87	44 / 44	100
**Inbred**	18 / 32	56	22 / 44	50
BALB/c	12 / 18	67	18 / 22	82
C3H	5 / 18	28	2 / 22	9
C57BL/6 (Ly5.2+)	1 / 18	6	1 / 22	5
NIH	-	-	1 / 22	5
**Outbred**	14 / 32	44	22 / 44	50
Swiss	12 / 14	86	20 / 22	91
CD-1	1 / 14	7	2 / 22	9
Swiss and CD-1	1 / 14	7	-	-
**In and outbred**	2 / 32	6	-	-
BALB/c and Swiss	1 / 2	50		
C3H and Swiss	1 / 2	50		
**Rat**	4 / 39	10	-	-
Wistar	4 / 4	100		
**Dog**	1 / 39	3	-	-

Before ARRIVE guidelines appeared, animal gender was not reported in 13% of papers (5 / 39) while females were more used than males (20 / 39 vs. 12 / 39) and both sexes were used in two papers. After ARRIVE guidelines, male and female animals were employed almost in the same proportion (16 / 44 vs. 20 / 44) and only one paper used both sexes in the same experiment. Within this period, sex information was not reported in 16% of papers (7 / 44) (Table 2).

**Table 2:** Details of animal gender used in papers.

	Before-ARRIVE		After-ARRIVE		
Sex	Ratio	%	Ratio	%	p-value
Male	12 / 39	31	16 / 44	36	0.81
Female	20 / 39	51	20 / 44	45	0.81
Male and female	2 / 39	5	1 / 44	2	-
Not specified	5 / 39	13	7 / 44	16	-

Basic details about animal age, weight and microbiological status and source (Tables 3 and 4) were not provided in almost half of the reviewed publications, both pre- or post publication of ARRIVE guidelines.

**Table 3:** Details of animal models for Chagas disease.

	Before-ARRIVE		After-ARRIVE		
Details given	Ratio	%	Ratio	%	p-value
Age range	25 / 39	64	26 ^a^/ 44	59	0.81
Weight range	26 / 39	67	20 / 44	45	0.85
Specified source	16 / 39	41	21 / 44	48	0.69

**Table 4:** Microbiological status from experimental animals.

	Before-ARRIVE		After-ARRIVE		
Microbiological status	Ratio	%	Ratio	%	p-value
Specific Pathogen Free (SPF)	4 / 39	10	3 / 44	7	0.86
Conventional	3 / 39	8	4 / 44	9	0.99
Not specified	32 / 39	82	37 / 44	84	0.99

Macroenvironmental information such as room temperature was detailed in nearly the same proportion before and after ARRIVE guidelines publication (10 / 39 vs. 16 / 44), but reporting of light/dark cycle increased from 23% (9 / 39) to 43% (19 / 44) respectively, although not statistically significant.

In addition, more than half of the analyzed studies in both periods failed to mention any macroenvironmental parameters or included ambiguous information. However, the percentage decreased from 69% (27 / 39) to 57% (25 / 44) after publication of ARRIVE guidelines (Table 5), but this change was not enough to reach statistical significance.

**Table 5:** Macroenvironmental details.

	Before-ARRIVE		After-ARRIVE		
Macroenvironment details	Ratio	%	Ratio	%	p-value
Temperature	10 / 39	26	16 / 44	36	0.42
Humidity	0 / 39	0	1 / 44	2	0.99
Light/Dark cycle	9 / 39	23	19 / 44	43	0.89
Not specified ^a^	27 / 39	69	25 / 44	57	0.35

^a^ Includes statements as "standard conditions", "controlled environment", "temperature-controlled room" and one which refers to a previous published paper.

Concerning microenvironmental conditions, access to food and water (mostly *ad libitum*), was reported in same proportion (17 / 39 vs. 21 / 44) in papers published before or after ARRIVE guidelines appeared. Similarly to macroenvironmental details, more than half of the analyzed studies (22 / 39 and 27 / 44) failed to provide any microenvironmental information for both periods of time (Table 6).

**Table 6:** Microenvironment details.

	Before-ARRIVE		After-ARRIVE		
Microenvironment details	Ratio	%	Ratio	%	p-value
Type of cage	4 / 39	10	0 / 44	0	0.09
Bedding material	1 / 39	3	1 / 44	2	0.99
# of cage companions	7 / 39	18	11^a^ / 44	25	0.61
Access to food and water	17 / 39	44	21 / 44	48	0.88
Not specified ^b^	22 / 39	56	27 / 44	61	0.81

^a^ Includes 2 papers which declares individual cages for better management.

^b^ Includes statements as "standard conditions" and one which refers to a previous published paper.

### Information about ethical statement and 3R’s Principles

Before ARRIVE guidelines became widely available, approximately 51% of papers on animal models evaluating drugs for Chagas disease included a statement about compliance with local or international guidelines for experimentation with animals. This percentage increased to 66% (p = 0.26) after ARRIVE guidelines were published.

Reporting of protocol revision and approval by an Institutional Animal Care and Use Committee (IACUC) increased from 26% (10 / 39) to 41% (18 / 44) after ARRIVE guidelines publication (p = 0.22) (Table 7).

**Table 7:** Ethical statements in animal models for Chagas disease.

	Before-ARRIVE		After-ARRIVE		
Ethical statements	Ratio	%	Ratio	%	p-value
Refers to guidelines ^a^	20 / 39	51	29 / 44	66	0.26
Protocol approbation without number of permission	11 / 39	28	15 / 44	34	0.73
Protocol approbation with number of permission	10 / 39	26	18 / 44	41	0.22

^a^ National or international guidelines.

The number of papers that mentioned refined strategies and procedures increased from 15% (6 / 39) to 23% (10 / 44) after ARRIVE guidelines publication. Ten publications mentioned proceedings which would require anesthesia/analgesia (such as terminal bleeding. bioluminescence imaging techniques); seven of these reports (70%) described the procedures only as “under anesthesia” or “with anesthetized mouse” without further details, while two of them specifically reported isofluorane use.

The vast majority of the reviewed papers failed to mention euthanasia methods. Some papers published before ARRIVE guidelines applied methods not accepted nowadays; only three reported carbon dioxide use, all of them published after the ARRIVE guidelines (Table 8).

**Table 8:** Welfare-related assessments.

	Before-ARRIVE		After-ARRIVE		
Welfare-related assessments	Ratio	%	Ratio	%	p-value
Anesthesia, analgesia ^a^	8 / 16	50	6 / 10	60	0.93
Refinement strategies	6 ^b^ / 39	15	10 ^c^ / 44	23	0.57
Euthanasia method	6 ^d^ / 39	15	3 ^e^ / 44	7	0.37

^a^ Applied for non-invasive procedures (electrocardiogram, bioluminescence imaging) or for terminal bleeding.

^b^ Includes 3 days to acclimation before study star/ and statement to follow 3R's principle.

^c^ Includes anticipated end points in parasitaemia peak or with severe adverse effects, refined oral administration or oral volume up to 50 μL/animal or 7 to 10 days to acclimation before study starts.

^d^ Methods: tribromoethanol followed by decapitation; dyethyl-ether followed by cervical dislocation; thiopental overdose; CO2 inhalation.

^e^ CO2 inhalation in all cases.

### Reported experimental design and sample size calculation data

The ARRIVE guidelines checklist includes statements about reporting of sample size calculations and statistical methods used. More than 60% (27 / 39 and 27 / 44) of the reviewed papers, all published after ARRIVE guidelines, reported the statistical tests used to analyze results, but only 11% (5 / 44) gave information about data distribution,

Regarding experimental design, a similar proportion of papers from both periods (5 / 39 and 7 / 44) declared treatment randomization or any effort to minimize subjective bias (*e*.*g*. randomized block design). None of the publications in any studied period substantiated the sample size employed (Table 9).

**Table 9:** Experimental design in animal models for Chagas disease.

* *	Before-ARRIVE		After-ARRIVE		
Experimental design	Ratio	%	Ratio	%	p-value
Treatment randomization or steps to minimize subjective bias	5 ^a^ / 39	13	7 / 44	16	0.93
Sample size calculation	0 / 39	0	0 / 44	0	0.99
Methods used to assess data distribution	0 / 39	0	5 ^b^ / 44	11	0.057
Statistical methods used for analysis	27 / 39	69	27 / 44	61	0.50

^a^ Includes one randomized blocking design by cage (one replication for each treatment group in same cage).

^b^ Includes one paper which refers to "homogeneous" or "heterogeneous" data.

### Animal models for Chagas disease

Tables 10–16 show different characteristics of reported animal models of *T*. *cruzi* infection.

**Table 10:** Experimental characteristics in animal models for Chagas disease.

Experimental infection model	Before-ARRIVE		After-ARRIVE	
Phase disease under treatment	Ratio	%	Ratio	%
Acute phase	37 / 39	94	33 / 44	75
Sub acute phase ^a^	-	-	1 / 44	2
Chronic phase	1 / 39	3	1 / 44	2
Acute and chronic phase ^b^	1 / 39	3	9 ^c^ / 44	20

^a^ Defined as 40 dpi.

^b^ Treatment at both phases in same paper.

^c^ Includes 2 papers which do not treat in chronic phase, only antibody follow up.

**Table 11:** *T*. *cruzi* strains employed in animal models for Chagas disease.

	Before-ARRIVE		After-ARRIVE	
*T*. *cruzi* strain	Ratio	%	Ratio	%
Y	15 / 39	38	12 ^a^ / 44	27
Tulahuén	6 / 39	15	8 ^b^ / 44	18
H4	1 / 39	3	4 / 44	9
CL Brener ^c^	2 / 39	5	-	-
CA-I/72	2 / 39	5	-	-
Maracay	3 / 39	8	-	-
SN3	-	-	5 / 44	11
Brazil	-	-	2 / 44	5
More than one strain in study	5 / 39	13	8 / 44	18
Other strain	4 / 39	13	5 / 44	11

^a^ Includes transfected Y-luciferase.

^b^ Includes Tulahuén 20A clone.

^c^ Includes CL Brener-clone 5.

**Table 12:** Inoculum size for experimental animal models for Chagas disease.

Inoculum size ^a^	Before-ARRIVE		After-ARRIVE	
For acute phase model	Ratio	%	Ratio	%
<1.000	4 / 39	10	5 / 40	3
1.000–5.000	11 / 39	28	11 / 40	23
10.000–50.000	10 / 39	26	23 / 40	53
≥100.000	6 / 39	15	9 / 40	23
2000/kg	1 / 39	3	-	-
**For chronic phase model**				
<1.000	1 / 39	3	2 / 11	18
1.000–5.000	-	-	3 / 11	27
10.000–50.000	-	-	2 / 11	18
≥100.000	-	-	4 /11	36
**Different inoculum in same work**	6 / 36	17	4 / 44	9

^a^ trypomastigotes/animal

**Table 13:** Inoculation route of T. cruzi in animal models for Chagas disease.

	Before-ARRIVE		After-ARRIVE	
Inoculation route	Ratio	%	Ratio	%
Intraperitoneal	31 / 39	87	42 / 44	95
Intradermal	3 / 39	8	-	-
Subcutaneous	1 / 39	3	-	-
Not specified	1 / 39	3	2 / 44	5

**Table 14:** Treatment route in animal models for Chagas disease.

	Before-ARRIVE		After-ARRIVE	
Treatment route	Ratio	%	Ratio	%
Oral	13 / 39	33	22 / 44	50
IP	14 / 39	36	11 / 44	25
Oral–Intraperitoneal	4 / 39	10	8 / 44	18
Oral *ad-libitum* ^a^	-	-	2 / 44	5
Oral -Subcutaneous	2 / 39	5	-	-
IV	4 / 39	10	1 / 44	2
SC	2 / 39	5	-	-
Not specified	-	-	-	-

^a^ in water/food.

**Table 15:** Treatment initiation in animal models for Chagas disease.

	Before-ARRIVE		After-ARRIVE	
Treatment initiation	Ratio	%	Ratio ^a^	%
0 to 48 hs after infection	16 / 39	41	10 / 51	20
At parasitaemia onset	13 / 39	33	32 / 51	63
At subacute phase ^b^	-	-	1 / 51	2
Chronic treatment ^c^	1 / 39	3	4 / 51	8
Not specified	3 / 39	8	2 / 51	4
Various schemes	6 / 39	15	-	-
Before infection ^d^	-	-	2 / 51	4

^a^ Over a total of 51 models employed.

^b^ Defined at 40 dpi.

^c^ Defined at 60, 75, 90 or 120 dpi.

^d^ 14 days or 24 hours pre infection.

**Table 16:** Treatment length and schedule in animal models for Chagas disease.

Length of treatment	Ratio	%	Ratio	%
180 consecutive days	**-**	**-**	1 / 51	2
90 consecutive days	2 / 39	5	**-**	**-**
60 consecutive days	1 / 39	3	2 / 51	4
35 consecutive days	**-**	**-**	2 / 51	4
30 consecutive days	4 / 39	10	4 / 51	8
28 consecutive days	1 / 39	3	2 / 51	4
21 consecutive days	3 / 39	8	**-**	**-**
20 consecutive days	6 / 39	15	11 / 51	22
15 consecutive days	2 / 39	5	2 / 51	4
14 consecutive days	3 / 39	8	**-**	**-**
13 consecutive days	1 / 39	3	1 / 51	2
10 consecutive days	1 / 39	3	**-**	**-**
7 consecutive days	**-**	**-**	1 / 51	2
5 consecutive days	4 / 39	10	9 / 51	18
*Ad libitum* in water or chow	**-**	**-**	2 / 51	4
Different schedules	7 / 39	18	6 / 51	12
60 doses	1 ^a^ / 39	3	**-**	**-**
10 doses	1 ^b^ / 39	3	**-**	**-**
5 doses	1 ^c^ / 39	3	-	-
2 doses	-	**-**	1 ^d^ / 51	2
1 dose at the peak	-	**-**	1 ^e^ / 51	2
1 dose at 3 dpi	1 / 39	3	-	-

^a^ 1 daily dose, 6 days/week, for a total of 60 doses.

^b^ Consecutive doses or every other day.

^c^ 3 consecutives doses every 24 hs, and 2 doses every 24 hs after 24 hs.

^d^ At 5 and 8 dpi.

^e^ At 19 or 24 dpi.

To assess efficacy of new compounds, acute infection was the preferred phase to start treatment, both before and after ARRIVE guidelines publication. In only 1 (3%) and nine papers (20%), before and after ARRIVE respectively, drugs were tested in both acute and chronic stage in separated essays. No rationale was provided for studying parasiticidal effects of drugs in chronic animal models.

Most used *T*. *cruzi* strains were Y strain and Tulahuen, in 38 and 27% (before ARRIVE) and 15 and 18% papers (after ARRIVE), respectively. Furthermore, thirteen publications in total reported assessing compound efficacy on more than one *T*. *cruzi* strain.

A wide range of inoculum sizes were reported, from less than 1,000 to more than 100,000 trypomastigotes per animal. Inoculation was intraperitoneal in the vast majority of papers (87 and 95%, before and after ARRIVE respectively), whilst 3 to 5% of papers did not specify such information.

Before ARRIVE guidelines publication, treatment was administered most commonly by oral (13 / 39) or intraperitoneal routes (14 / 39). After ARRIVE treatment was reportedly administered by the oral route in half of the reviewed papers, while eleven (25%) preferred the intraperitoneal route. Treatment initiation, schemes and duration were reported with large variations.

## Discussion

Research involving animal studies is essential to many disciplines in the biomedical sciences. Detailed descriptions in publications of experimental methods and results enable researchers to interpret data, evaluate results accurately, replicate findings and move science forward [9].

The “Materials and methods” section of research papers is intended to provide basic information about how the research was performed. Comprehensive reporting is essential to correctly understand how investigations were undertaken, to properly interpret findings [10] and to compare and integrate results obtained from previous experiments.

Consistent reporting of animal use is directly related to scientific quality. Employed animals should not be unnecessarily stressed and should be kept under appropriately controlled conditions: poor animal welfare is likely to result in poor science [11]. Moreover, experiments involving animals have also ethical requirements and are increasingly scrutinized by the public opinion.

Minimum information guidelines seek to promote transparency in experimental reporting, enhance accessibility to data and support effective quality assessment, which increases the general value of data, and therefore, of scientific evidence [7].

In this sense, some initiatives such as the Guidance for the Description of Animal Research in Scientific Publications by the National Research Council (NRC), the Gold Standard Publication Checklist (GSPC) [12] and the Animals in Research: Reporting i*n vivo* Experiments (ARRIVE) [8], have been published with the aim to be adopted as a requirement for publication.

For this review, ARRIVE guidelines were used as a benchmark to measure quality in animal use reporting use because of its wide acceptance, and its useful checklist to easily identify key information.

Chagas disease is one of the seventeen neglected disease prioritized by World Health Organization and a secure and effective treatment is urgently needed. Despite high throughput screening systems and growing capacity to identify anti-*T*. *cruzi* compounds, both from pharmaceutical companies’ libraries and the public domain, many lead compounds with promissory results in animals models of infection have unfortunately failed in clinical trials [3,13].

To evaluate the quality of information reported in articles referred to new compounds for Chagas disease, we contrasted descriptions of animal use and care with those descriptions suggested by the ARRIVE Guidelines, using the guidelines checklist. In order to compare the quality of report before and after the ARRIVE guidelines publication, the date period for the search was selected from 2008/06/30 to 2014/06/30, a year after ARRIVE guidelines first appearance in print.

We observed that before publication of the ARRIVE guidelines, animal species used as models for experimental infection with *T*. *cruzi* seemed more diverse, even though mice were the most commonly employed. After ARRIVE publication, *Mus musculus* was the only species used to assess efficacy of new chemotherapies for Chagas disease in the papers published. This election of animal model may be explained by the historical use of mice to evaluate new compounds [14,15] and by the conclusions reached at the Experimental Models in Drug Screening and Development for Chagas Disease workshop, held in Rio de Janeiro, Brazil in 2008, which suggested the use of the Mouse model [16]. However, no justifications for the chosen animal models were provided in any of the papers reviewed.

There is possibly no ideal animal model to test drugs for Chagas disease (i.e. an exclusively Human disease), but some models may better mimic particular aspects of the disease [17]. Given that only animal species used after publication of ARRIVE guidelines, (i.e. the Mouse), may not resemble all Chagas disease stages and their complexity in the human host, other animal species with pathogenesis more similar to the Human (eg. Guinea pigs (*Cavia porcellus*)) [18] could be employed, depending on the aim of the research or whether encouraging results are obtained in a murine model of infection.

Among mice, we observed that inbred and outbred strains were employed in same proportion. Strain selection is a crucial decision due differences and variability in response.

Inbred strains are genetically defined and frequently stable, homogenous, and more often lead to repeatable outcomes than ‘‘genetically undefined” outbred stocks. Experiments with Mouse inbred strains may be more powerful with more accurate dose-response relationships and fewer false negative results than those carried out using outbred stocks [19].

Outcomes in animal models of *T*. *cruzi* infection are dependent on many factors, including animal species, strain, age, sex, *T*. *cruzi* strain, inoculum size and route of infection, among others [20]. Since several variables contribute to establish an *in vivo* model, reported information must be as detailed as possible.

In the papers reviewed from the period after ARRIVE publication, information on animal gender was not provided in seven publications (16%), a proportion similar to that observed before ARRIVE guidelines publication (13%). In a similar previous survey, Kilkenny et al. revealed that in 24 of 72 reviewed papers (33%) sex was not reported [21]. This observed lack of detail in reporting undermines repeatability and robustness of studies, since some research suggests that response to infection in male mice is different from females which are, apparently, more resistant to *T*. *cruzi* infection [22,23]. Interestingly, a few of the reviewed papers used both sexes, but did not analyze results with a factorial design missing the opportunity to test for interactions between factors (sex) and drug response or disease progression [23–25].

Regrettably, other key information such as age and weight range at time of infection was missing in more than the half of the publications both before and after the ARRIVE guidelines publication. This omission prevents further testing of covariates, if necessary, as these variables may modify the final outcome of certain models.

Animal source is not reported in more than 50% of papers, irrespective of period studied; This, added to the lack of information on animal microbiological status, goes clearly in detriment of quality standards of any preclinical studies. Besides, results can be distorted and misunderstood by concurrent infections, as reported previously in a biological characterization of a *T*. *cruzi* strain [26].

Macro and microenvironment are essential variables which influence animal well-being and, accordingly, repeatability and reproducibility of the results. This information was incomplete or totally absent in more than half of the papers from both periods reviewed. ARRIVE guidelines do not seem to have had an impact on reporting of these variables since there were no significant differences between information provided in articles published before or after ARRIVE release. For instance, exposure to wide extremes temperature may result in behavioral, physiologic, and morphologic changes, which might negatively affect animal well-being and research performance as well as outcomes of research protocols. [27]. The standard temperature range for mice and other rodents is 23 ± 3°C to prevent triggering compensatory thermoregulatory mechanisms that can affect animal health, and alter experimental results. A good management program provides environment, housing, and care that minimizes variations that can affect research [28]. Unfortunately, more than half of the papers evaluated from both periods did not report any information about macroenvironmental conditions. Similarly, more than 50% of publications did not report any information relative to microenvironment variables. These factors can potentially influence experimental results and are therefore scientifically important so it is unclear why omission of these essential details is so prevalent.

Two papers admitted to housing animals in individual cages, “for better management”. This can make workload easier for personnel, but when it comes to animal welfare, individual caging can be more harmful since solitary confinement may increase alter immunological responses, produces changes in body and organ weights and alterations in blood cell counts, among others, potentially affecting drug response [28]. Adding to this, there is a growing concern in public opinion about animal laboratory testing and a well-detail husbandry conditions may contribute to proper understanding even to lay public. What is written in those reports, and how it is written, may thus be crucial to the public perception of animal experiments [11].

Investigators conducting research with animal subjects have an ethical and legal responsibility to ensure they are treated humanely. Scientists are required to conduct their studies in compliance with a framework of federal, state, local, and institutional rules and regulations [29].

It is widely accepted that applying 3R’s Principles to experiments using animals is in consonance with good scientific practice [21]. Since there is no validated replacement method yet to assess efficacy and safety compounds for Chagas disease treatment in humans, animals models are expected to fill the gap between *in vitro* testing and clinical trials. Therefore, strategies to refine procedures and reduce pain and distress are desired.

Typically, parasitaemia values and mortality were the principal outcomes used to assess trypanocidal activity in the papers reviewed. Times have changed and it is currently necessary to establish and validate anticipated endpoints (i.e. endpoints that can predict death and can be used to avoid unnecessary suffering or distress in the experimental animals), which carries benefit for both researchers (e.g. they do not lose samples for histopathology, sera, etc) and animals (e.g. avoiding stressing death as result of sickness behavior and septic shock) [30].

At any rate, only 14% (5 / 36) and 23% (10 / 44) of papers (before and after ARRIVE guidelines publications, respectively) applied any refinement strategy, including anticipated endpoints at parasitaemia peak or with severe adverse effects, with a clear, but unjustifiably modest, increment in papers appeared after ARRIVE guidelines arise.

A comprehensive analysis of experimental design and statistical methods is beyond the scope of this review, but many topics recommended in the ARRIVE guidelines are missing or incomplete from analyzed publications,. Treatment randomization, an essential step to avoid experimental bias, was declared only in 16% (7 / 44) publications.

Sample size was not justified in any of the papers, suggesting that there was no previous sample size calculation, and that animal numbers were more a matter of habit than a statistical decision. One may argue that is difficult to predict parasitaemia levels in this models, and that dispersion is very large (due in part to direct counting methods in Neubauer chamber or between glass side and cover slip) which would make determining accurate sample size difficult. Nevertheless, strategies exists to justify number of animals employed such as conducting previous pilot studies, or applying Mead’s resource equation, suitable in cases where there is no information about standard deviation and/or it is difficult to specify an effect size [31,32].

These results agree with a previous quality of reporting survey which observed that only 5 in 72 (7%) of studies using mice informed sample size calculations or treatment randomization [21].

Statistical methods were declared and detailed in nearly 2/3 of publications, but since there was no information about data distribution, a proper analysis of the correct application of these methods cannot be established.

As result from a workshop held in 2008 [16], guiding principles for drug testing in animal models of Chagas disease were put forward, in which certain experimental variables were agreed upon in order to perform similar research in different groups, allowing to screen candidate compounds and discard or move forward rapidly compounds to further testing.

Although original standardized protocols could be modified and updated, the initiative was very promissory but only partially accepted by the Chagas scientific community given the variety of existing animal models using different mice and parasite strains, inoculums sizes, treatment schedules and others differing factors. Unfortunately, it seems that standardization of animal models is no easy in the field, possibly due to difficulties in accessing animal and/or parasite strains from different those already in use by each group. The fact that parasite strains in particular, are not easy (or cheap) to transfer across country borders, among other issues, should be kept in mind when judging this difficulties.

Finally, regarding suggestions put forward by Romanha et al., only 50% of the publications described the use of oral treatment (as suggested), and 63% of the studies started treatment with patent parasitaemia. Also, less than one-fourth (22%) of studies performed a treatment for at least 20 consecutive days, indicating an incomplete and partial adherence to the suggested guidelines for *in vivo* drug screening for Chagas disease [16].

Our results illustrate a general lack of compliance with ARRIVE guidelines in research involving animals for testing of efficacy of new compounds for Chagas disease treatment. Other fields in preclinical research are not exempt from these problems, according to conclusions obtained in a survey conducted in experimental autoimmune encephalomyelitis and multiple sclerosis two years before ARRIVE publication [33]. Unfortunately, we observed that publication of clear guidelines such as ARRIVE was not sufficient to improve reporting of animal studies, at least in the field of Chagas disease drug research.

In conclusion, a systematic review has been carried out to measure adherence degree to ARRIVE guidelines in animal models for new chemotherapy for Chagas disease treatment. There is vast key information missed or incomplete which difficult proper evaluation and comprehension of obtained results. Ensuring animal well-being and responsible use while meeting scientific aims must be emphasize to allow translational research to contribute to resolve affected population problems.

This review does not want to cast doubt the results obtained in the evaluated papers, and is not its matter examine their scientific merits. On the contrary, it attempts to warn about the weak reporting quality in search of new chemotherapy compounds for Chagas disease and has a teaching intention to encourage scientific community to adopt ARRIVE guidelines to correctly report their preclinical trial results and to unify animal models in order to maximize obtained information and to be more transparent inside and outside the academic field.

We did not observe an improvement in publication quality after ARRIVE guidelines publication, compared to the previous period. There is a clear need to improve design and reporting of animal research studies in Chagas disease. Full compliance with ARRIVE guidelines would be a welcome starting point.

## Supporting Information

S1 Checklist PRISMA Checklist. (DOC)

S1 Fig Number of papers by publication year. (DOCX)

S2 Fig Search filter before and after ARRIVE guidelines. (DOCX)

S3 Fig Flow diagram. (DOC)

S1 Text Supporting References. (DOCX)
