# Supplementary material for: Quality of Reporting and Adherence to ARRIVE Guidelines in Animal Studies for Chagas Disease Preclinical Drug Research: A Systematic Review
Source: PLoS Negl Trop Dis. 2015 Nov 20;9(11):e0004194. doi: 10.1371/journal.pntd.0004194 (PMC4654562; doi:10.1371/journal.pntd.0004194)
Supplement: S1 Fig — (DOCX) [file pntd.0004194.s002.docx]

**Supplementary Material. S1. Number of papers by publication year.**

| **Publication year** | **Ratio** | **%** | **Publication year** | **Ratio** | **%** |
| --- | --- | --- | --- | --- | --- |
| **Before jun-11** | 6 / 39 | 15 | **2014** | 6 / 44 | 14 |
| **2010** | 19 / 39 | 49 | **2013** | 14 / 44 | 31 |
| **2009** | 9 / 39 | 23 | **2012** | 18 / 44 | 41 |
| **2008** | 5 / 39 | 13 | **After jul-11** | 6 / 44 | 14 |
