# Supplementary material for: Quality of Reporting and Adherence to ARRIVE Guidelines in Animal Studies for Chagas Disease Preclinical Drug Research: A Systematic Review
Source: PLoS Negl Trop Dis. 2015 Nov 20;9(11):e0004194. doi: 10.1371/journal.pntd.0004194 (PMC4654562; doi:10.1371/journal.pntd.0004194)
Supplement: S3 Fig — (DOC) [file pntd.0004194.s004.doc]

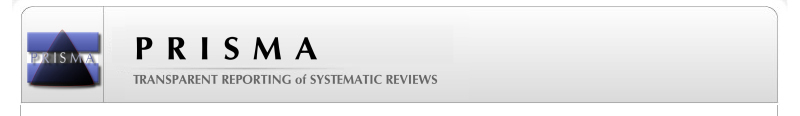
**PRISMA 2009 Flow Diagram**

**Screening**

**Included**

**Eligibility**

**Identification**

Records identified through database searching
(n =305)

Additional records identified through other sources
(n =0)

Records after duplicates removed
(n = 305)

Records screened
(n =305)

Records excluded
(n =222)

Full-text articles assessed for eligibility
(n=83)

Full-text articles excluded, with reasons
(n 0)

Studies included in qualitative synthesis
(n=83)

Studies included in quantitative synthesis (meta-analysis)
NA

(not applicable)
