# Supplementary material for: Quality of Reporting and Adherence to ARRIVE Guidelines in Animal Studies for Chagas Disease Preclinical Drug Research: A Systematic Review
Source: PLoS Negl Trop Dis. 2015 Nov 20;9(11):e0004194. doi: 10.1371/journal.pntd.0004194 (PMC4654562; doi:10.1371/journal.pntd.0004194)
Supplement: S1 Text — (DOCX) [file pntd.0004194.s005.docx]

1. Aleixo D, Ferraz F, Ferreira E, de Lana M, Gomes M, de Abreu Filho B, et al. Highly diluted medication reduces parasitemia and improves experimental infection evolution by *Trypanosoma cruzi*. BMC Res Notes. 2012;5. doi:10.1186/1756-0500-5-352

2. Andriani G, Chessler A, Courtemanche G, Burleigh B, Rodriguez A. Activity *in vivo* of anti-*Trypanosoma cruzi* compounds selected from a high throughput screening. PLoS Negl Trop Dis. 2011;5: e1298. doi:10.1371/journal.pntd.0001298

3. Arantes J, Francisco A, de Abreu Vieira P, Silva M, Araújo M, de Carvalho A, et al. *Trypanosoma cruzi*: desferrioxamine decreases mortality and parasitemia in infected mice through a trypanostatic effect. Exp Parasitol. Elsevier Inc.; 2011;128: 401–8. doi:10.1016/j.exppara.2011.05.011

4. Bahia M, de Andrade I, Martins T, do Nascimento Á, Diniz LF, Caldas I, et al. Fexinidazole: a potential new drug candidate for Chagas Disease. PLoS Negl Trop Dis. 2012;6: e1870. doi:10.1371/journal.pntd.0001870

5. Batista DG, Batista M, de Oliveira G, Britto C, Rodrigues A, Stephens C, et al. Combined treatment of heterocyclic analogues and benznidazole upon *Trypanosoma cruzi in vivo*. PLoS One. 2011;6: e22155. doi:10.1371/journal.pone.0022155

6. Batista DG, Batista M, de Oliveira G, do Amaral P, Lannes-Vieira J, Britto C, et al. Arylimidamide DB766, a potential chemotherapeutic candidate for Chagas’ disease treatment. Antimicrob Agents Chemother. 2010;54: 2940–2952. doi:10.1128/AAC.01617-09

7. Bazán P, Lo Presti M, Rivarola H, Triquell M, Fretes R, Fernández A, et al. Chemotherapy of chronic indeterminate Chagas disease: a novel approach to treatment. Parasitol Res. 2008;103: 663–9. doi:10.1007/s00436-008-1029-x

8. Boiani L, Davies C, Arredondo C, Porcal W, Merlino A, Gerpe A, et al. *In vivo* studies of 5-arylethenylbenzofuroxans in acute murine models of Chagas’ disease. Eur J Med Chem. 2008;43: 2229–37. doi:10.1016/j.ejmech.2007.12.016

9. Branquinho R, Mosqueira V, de Oliveira-Silva J, Simões-Silva M, Saúde-Guimarães D, de Lana M. Sesquiterpene lactone in nanostructured parenteral dosage form is efficacious in experimental Chagas disease. Antimicrob Agents Chemother. 2014;58: 2067–75. doi:10.1128/AAC.00617-13

10. Buckner F, Bahia M, Suryadevara P, White K, Shackleford D, Chennamaneni N, et al. Pharmacological Characterization, Structural Studies, and *in vivo* Activities of Anti-Chagas Disease Lead Compounds Derived from Tipifarnib. Antimicrob Agents Chemother. 2012;56: 4914–21. doi:10.1128/AAC.06244-11

11. Bustamante J, Craft J, Crowe B, Ketchie S, Tarleton R. New, combined and reduced dosing treatment protocols cure *Trypanosoma cruzi* infection in mice. J Infect Dis. 2014;209: 150–62. doi:10.1093/infdis/jit42

12. Caballero A, Marín C, Rodríguez-Diéguez A, Ramírez-Macías I, Barea E, Sánchez-Moreno M, et al. *In vitro* and *in vivo* antiparasital activity against *Trypanosoma cruzi* of three novel 5-methyl-1,2,4-triazolo[1,5-a]pyrimidin-7(4H)-one-based complexes. J Inorg Biochem. Elsevier Inc.; 2011;105: 770–6. doi:10.1016/j.jinorgbio.2011.03.015

13. Cabrera E, Murguiondo M, Arias M, Arredondo C, Pintos C, Aguirre G, et al. 5-Nitro-2-furyl derivative actives against *Trypanosoma cruzi*: Preliminary *in vivo* studies. Eur J Med Chem. 2009;44: 3909–14. doi:10.1016/j.jinorgbio.2011.03.015

14. Caetano L, Santello F, Del Vecchio Filipin M, Brazão V, Caetano L, Toldo M, et al. *Trypanosoma cruzi*: Dehydroepiandrosterone (DHEA) and immune response during the chronic phase of the experimental Chagas’ disease. Vet Parasitol. 2009;163: 27–32. doi:10.1016/j.vetpar.2009.03.053

15. Canavaci A, Bustamante J, Padilla A, Perez Brandan C, Simpson L, Xu D, et al. *In vitro* and *in vivo* High-Throughput Assays for the Testing of Anti-*Trypanosoma cruzi* Compounds. PLoS Negl Trop Dis. 2010;4: e740. doi:10.1371/journal.pntd.0000740

16. Cencig S, Coltel N, Truyens C, Carlier Y. Evaluation of benznidazole treatment combined with nifurtimox, posaconazole or AmBisome® in mice infected with *Trypanosoma cruzi* strains. Int J Atimicrob Agents. 2012;40: 527–32. doi:10.1016/j.ijantimicag.2012.08.002

17. Cencig S, Coltel N, Truyens C, Carlier Y. Parasitic Loads in Tissues of Mice Infected with *Trypanosoma cruzi* and Treated with AmBisome. PLoS Negl Trop Dis. 2011;5: e1216. doi:10.1371/journal.pntd.0001216

18. Chen Y, Brinen L, Kerr I, Hansell E, Doyle P, McKerrow J, et al. *In vitro* and *in vivo* Studies of the Trypanocidal Properties of WRR-483 against *Trypanosoma cruzi*. PLoS Negl Trop Dis. 2010;4: e825. doi:10.1371/journal.pntd.0000825

19. Ciccarelli A, Frank F, Puente V, Malchiodi E, Batlle A, Lombardo M. Antiparasitic Effect of Vitamin B12 on *Trypanosoma cruzi*. Antimicrob Agents Chemother. 2012;56: 5315–20. doi:10.1128/AAC.00481-12

20. da Silva Ferreira D, Esperandim V, Toldo M, Kuehn C, do Prado Júnior J, Cunha W, et al. *In vivo* activity of ursolic and oleanolic acids during the acute phase of *Trypanosoma cruzi* infection. Exp Parasitol. Elsevier Inc.; 2013;134: 455–9. doi:10.1016/j.exppara.2013.04.005

21. da Silva C, Batista DG, De Araújo J, Batista M, Lionel J, de Souza E, et al. Activities of psilostachyin A and cynaropicrin against *Trypanosoma cruzi* *in vitro* and *in vivo*. Antimicrob Agents Chemother. 2013;57: 5307–14. doi:10.1128/AAC.00595-13

22. da Silva C, Batista DG, Oliveira G, de Souza E, Hammer E, da Silva P, et al. *In vitro* and *in vivo* investigation of the efficacy of Arylimidamide DB1831 and its mesylated salt form -DB1965- against *Trypanosoma cruzi* Infection. PLoS One. 2012;7: e30356. doi:10.1371/journal.pone.0030356

23. da Silva C, Batista M, Batista DG, de Souza E, da Silva P, de Oliveira G, et al. *In vitro* and *in vivo* Studies of the Trypanocidal Activity of a Diarylthiophene Diamidine against *Trypanosoma cruzi*. Antimicrob Agents Chemother. 2008;52: 3307–14. doi:10.1128/AAC.00038-08

24. Davies C, Cardozo R, Negrette O, Mora M, Chung M, Basombrio M. Hydroxymethylnitrofurazone Is Active in a Murine Model of Chagas’ Disease. Antimicrob Agents Chemother. 2010;54: 3584–9. doi:10.1128/AAC.01451-09

25. de Paula Costa G, Silva R, Pedrosa M, Pinho V, de Lima W, Teixeira M, et al. Enalapril prevents cardiac immune-mediated damage and exerts anti- *Trypanosoma cruzi* activity during acute phase of experimental Chagas disease. Parasite Immunol. 2010;32: 202–8. doi:10.1111/j.1365-3024.2009.01179.x

26. Díaz-Chiguer D, Márquez-Navarro A, Nogueda-Torres B, de la Luz León-Ávila G, Pérez-Villanueva J, Hernández-Campos A, et al. *In vitro* and *in vivo* trypanocidal activity of some benzimidazole derivatives against two strains of *Trypanosoma cruzi*. Acta Trop. Elsevier B.V.; 2012;122: 108–12. doi:10.1016/j.actatropica.2011.12.009

27. Diniz LF, Caldas I, Guedes P, Crepalde G, de Lana M, Carneiro C, et al. Effects of ravuconazole treatment on parasite load and immune response in dogs experimentally infected with *Trypanosoma cruzi*. Antimicrob Agents Chemother. 2010;54: 2979–86. doi:10.1128/AAC.01742-09

28. Diniz LF, Urbina J, de Andrade I, Mazzeti A, Martins T, Caldas I, et al. Benznidazole and posaconazole in experimental Chagas disease: positive interaction in concomitant and sequential treatments. PLoS Negl Trop Dis. 2013;7: e2367. doi:10.1371/journal.pntd.0002367

29. Doyle P, Chen C, Johnston J, Hopkins S, Leung S, Jacobson M, et al. A Nonazole CYP51 Inhibitor Cures Chagas’ Disease in a Mouse Model of Acute Infection. Antimicrob Agents Chemother. 2010;54: 2480–8. doi:10.1128/AAC.00281-10

30. Esperandim V, da Silva Ferreira D, Rezende K, Cunha W, Saraiva J, Bastos J, et al. Evaluation of the *in vivo* therapeutic properties of (-)-cubebin and (-)-hinokinin against *Trypanosoma cruzi*. Exp Parasitol. Elsevier Inc.; 2013;133: 442–6. doi:10.1016/j.exppara.2012.12.005

31. Fauro R, Lo Presti S, Bazan C, Baez A, Strauss M, Triquell F, et al. Use of clomipramine as chemotherapy of the chronic phase of Chagas disease. Parasitology. 2013;140: 917–27. doi:10.1017/S0031182013000103

32. Ferreira DS, Esperandim V, Toldo M, Saraiva J, Cunha W, de Albuquerque S. Trypanocidal activity and acute toxicity assessment of triterpene acids. Parasitol Res. 2010;106: 985–9. doi:10.1007/s00436-010-1740-2

33. Ferreira M, Cebrián-Torrejón G, Corrales A, Vera de Bilbao N, Rolón M, Gomez C, et al. Zanthoxylum chiloperone leaves extract: First sustainable Chagas disease treatment. J Ethnopharmacol. Elsevier Ireland Ltd; 2011;133: 986–93. doi:10.1016/j.jep.2010.11.032

34. Francisco A, de Abreu Vieira P, Arantes J, Pedrosa M, Martins H, Silva M, et al. *Trypanosoma cruzi*: Effect of benznidazole therapy combined with the iron chelator desferrioxamine in infected mice. Exp Parasitol. Elsevier Inc.; 2008;120: 314–9. doi:10.1016/j.exppara.2008.08.002

35. Frare E, Santello F, Caetano L, Caldeira J, Toldo M, Prado JJ. Growth hormones therapy in immune response against *Trypanosoma cruzi*. Res Vet Sci. Elsevier Ltd; 2010;88: 273–8. doi:10.1016/j.rvsc.2009.10.001

36. Gobbi P, Baez A, Lo Presti M, Fernández A, Enders J, Fretes R, et al. Association of clomipramine and allopurinol for the treatment of the experimental infection with *Trypanosoma cruzi*. Parasitol Res. 2010;107: 1279–83. doi:10.1007/s00436-010-2002-z

37. Grosso N, Alarcon M, Bua J, Laucella S, Riarte A, Fichera L. Combined treatment with benznidazole and allopurinol in mice infected with a virulent *Trypanosoma cruzi* isolate from Nicaragua. Parasitology. 2013;140: 1225–33. doi:10.1017/S0031182013000176

38. Guedes P, Oliveira F, Gutierrez F, da Silva G, Rodrigues G, Bendhack L, et al. Nitric oxide donor trans-[RuCl([15]aneN4)NO]2+ as a possible therapeutic approach for Chagas’ disease. Br J Pharmacol. 2010;160: 270–82. doi:10.1111/j.1476-5381.2009.00576.x

39. Gulin J, Eagleson M, Postan M, Cutrullis R, Freilij H, Garcia-Bournissen F, et al. Efficacy of voriconazole in a murine model of acute *Trypanosoma cruzi* infection. J Antimicrob Chemother. 2013;68: 888–94. doi:10.1093/jac/dks478

40. Gusmão A, Castanho R, Andrade R, Farsetti C, Mathias A, Therezo A, et al. Vitamin C effects in mice experimentally infected with *Trypanosoma cruzi* QM2 strain. Rev Soc Bras Med Trop. 2012;45: 51–4. doi:10.1590/S0037-86822012000100010

41. Jimenez-Coello M, Acosta-Viana K, Guzman-Marin E, Perez Gonzalez C, Salud Perez Gutierrez M. Anti-trypanosomal activity of (8-hydroxymethylen)-trieicosanyl acetate against infective forms of *Trypanosoma cruzi*. Pharm Biol. 2010;48: 666–71. doi:10.3109/13880200903241853

42. Jiménez-Coello M, Acosta-Viana K, Salud Perez Gutierrez M, Guzmán-Marín ES. *In vivo* activity of (8-hydroxymethylen)-trieicosanyl acetate against *Trypanosoma cruzi* during acute phase of the infection. Afr J Tradit Complement Altern Med. 2011;8: 198–207. doi:10.4314/ajtcam.v8i5S.26

43. Jiménez-Coello M, Guzman-Marín E, Ortega-Pacheco A, Perez-Gutiérrez S, Acosta-Viana K. Assessment of the anti-protozoal activity of crude Carica papaya seed extract against *Trypanosoma cruzi*. Molecules. 2013;18: 12621–32. doi:10.3390/molecules181012621

44. Jimenez-Coello M, Guzman-Marin E, Perez-Gutierrez S, Polanco-Hernandez G, Acosta-Viana K. Antitrypanosomal activity of Senna villosa in infected BALB/c mice with *Trypanosoma cruzi* during the sub acute phase of infection. Afr J Tradit Complement Altern Med. 2011;8: 164–9. doi:10.4314/ajtcam.v8i5S.21

45. Keenan M, Abbott M, Alexander P, Armstrong T, Best W, Berven B, et al. Analogues of fenarimol are potent inhibitors of *Trypanosoma cruzi* and are efficacious in a murine model of Chagas disease. J Med Chem. 2012;55: 4189–204. doi:10.1021/jm2015809

46. Keenan M, Alexander P, Chaplin J, Abbott M, Diao H, Wang Z, et al. Selection and optimization of hits from a high-throughput phenotypic screen against *Trypanosoma cruzi*. Futur Med Chem. 2013;5: 1733–52. doi:10.4155/fmc.13.139

47. Keenan M, Alexander P, Diao H, Best W, Khong A, Kerfoot M, et al. Design, structure-activity relationship and *in vivo* efficacy of piperazine analogues of fenarimol as inhibitors of *Trypanosoma cruzi*. Bioorg Med Chem. 2013;21: 1756–63. doi:10.1016/j.bmc.2013.01.050

48. Keenan M, Chaplin J, Alexander P, Abbott M, Best W, Khong A, et al. Two analogues of fenarimol show curative activity in an experimental model of Chagas disease. J Med Chem. 2013;56: 10158–70. doi:10.1021/jm401610c

49. Kraus J, Verlinde C, Karimi M, Lepesheva G, Gelb M, Buckner F. Rational Modification of a Candidate Cancer Drug for Use Against Chagas Disease. J Med Chem. 2009;52: 9492–9. doi:10.1021/jm801313t

50. Kuehn C, Rodrigues Oliveira L, Santos C, Ferreira D, Alonso Toldo M, de Albuquerque S, et al. Melatonin and dehydroepiandrosterone combination: does this treatment exert a synergistic effect during experimental *Trypanosoma cruzi* infection? J Pineal Res. 2009;47: 253–9. doi:10.1111/j.1600-079X.2009.00708.x

51. Marim R, Gusmão A, Castanho R, Deminice R, Therezo A, Jordão Júnior A, et al. Effects of vitamin C supplementation on acute phase Chagas disease in experimentally infected mice with *Trypanosoma cruzi* QM1 strain. Rev Inst Med Trop Sao Paulo. 2012;54: 319–23.

52. Marín C, Ramírez-Macías I, López-Céspedes A, Olmo F, Villegas N, Díaz J, et al. *In vitro* and *in vivo* trypanocidal activity of flavonoids from Delphinium staphisagria against Chagas disease. J Nat Prod. 2011;74: 744–50. doi:10.1021/np1008043

53. Matsuo A, Silva L, Torrecilhas A, Pascoalino B, Ramos T, Rodrigues E, et al. *In vitro* and *in vivo* Trypanocidal Effects of the Cyclopalladated Compound 7a, a Drug Candidate for Treatment of Chagas’ Disease. Antimicrob Agents Chemother. 2010;54: 3318–25. doi:10.1128/AAC.00323-10

54. Miguel D, Ferraz M, Alves RO, Yokoyama-Yasunaka J, Torrecilhas A, Romanha A, et al. The anticancer drug tamoxifen is active against *Trypanosoma cruzi* *in vitro* but ineffective in the treatment of the acute phase of Chagas disease in mice. Mem Inst Oswaldo Cruz. 2010;105: 945–8. doi:10.1590/S0074-02762010000700021

55. Moreira D, Costa S, Hernandes M, Rabello M, de Oliveira Filho G, de Melo C, et al. Structural investigation of Anti- *Trypanosoma cruzi* 2- Iminothiazolidin-4-ones allows the identification of agents with efficacy in infected mice. J Med Chem. 2012;55: 10918–36. doi:10.1021/jm301518v

56. Nagajyothi F, Zhao D, Weiss L, Tanowitz H. Curcumin treatment provides protection against *Trypanosoma cruzi* infection. Parasitol Res. 2012;110: 2491–9. doi:10.1007/s00436-011-2790-9.Curcumin

57. Ndao M, Beaulieu C, Black W, Isabel E, Vasquez-Camargo F, Nath-Chowdhury M, et al. Reversible cysteine protease inhibitors show promise for a Chagas disease cure. Antimicrob Agents Chemother. 2014;58: 1167–78. doi:10.1128/AAC.01855-13

58. Olivieri B, Molina J, de Castro S, Pereira M, Calvet C, Urbina J, et al. A comparative study of posaconazole and benznidazole in the prevention of heart damage and promotion of trypanocidal immune response in a murine model of Chagas disease. Int J Antimicrob Agents. 2010;36: 79–83. doi:10.1016/j.ijantimicag.2010.03.006

59. Olmo F, Rotger C, Ramírez-Macías I, Martínez L, Marín C, Carreras L, et al. Synthesis and biological evaluation of N,N’-squaramides with high *in vivo* efficacy and low toxicity: toward a low-cost drug against Chagas disease. J Med Chem. 2014;57: 987–99. doi:10.1021/jm4017015

60. Papadopoulou M, Bloomer W, Rosenzweig H, Ashworth R, Wilkinson S, Kaiser M, et al. Novel 3-nitro-1H-1,2,4-triazole-based compounds as potential anti-Chagasic drugs: *in vivo* studies. Futur Med Chem. 2013;5: 1763–76. doi:10.4155/fmc.13.108.Novel

61. Polanco-Hernández G, Escalante-Erosa F, García-Sosa K, Acosta-Viana K, Chan-Bacab M, Sagua-Franco H, et al. *In vitro* and *in vivo* trypanocidal activity of native plants from the Yucatan Peninsula. Parasitol Res. 2012;110: 31–5. doi:10.1007/s00436-011-2447-8

62. Pupulin A, Marques-Araujo S, Toledo M, Gomes M, Takejima E, Cuman R, et al. Canova medication modifies parasitological parameters in mice infected with *Trypanosoma cruzi*. Exp Parasitol. Elsevier Inc.; 2010;126: 435–40. doi:10.1016/j.exppara.2010.04.002

63. Ramírez-Macías I, Marín C, Chahboun R, Messouri I, Olmo F, Rosales M, et al. *In vitro* and *in vivo* studies of the trypanocidal activity of four terpenoid derivatives against *Trypanosoma cruzi*. Am J Trop Med Hyg. 2012;87: 481–8. doi:10.4269/ajtmh.2012.11-0471

64. Ramírez-Macías I, Marín C, Es-Samti H, Fernández A, Guardia J, Zentar H, et al. Taiwaniaquinoid and abietane quinone derivatives with trypanocidal activity against T. cruzi and Leishmania spp. Parasitol Int. 2012;61: 405–13. doi:10.1016/j.parint.2012.02.001

65. Ramos E, Garza K, Krauth-Siegel R, Bader J, Martinez L, Maldonado R. 2,3-diphenyl-1,4-naphthoquinone: a potential chemotherapeutic agent against *Trypanosoma cruzi*. J Parasitol. 2009;95: 461–6. doi:10.1645/GE-1686.1

66. Rodrigues G, Feijó D, Bozza M, Pan P, Vullo D, Parkkila S, et al. Design, synthesis, and evaluation of hydroxamic acid derivatives as promising agents for the management of Chagas disease. J Med Chem. 2014;57: 298–308. doi:10.1021/jm400902y

67. Salomão K, de Souza E, Carvalho S, da Silva E, Fraga C, Barbosa H, et al. *In vitro* and *in vivo* Activities of 1,3,4-Thiadiazole-2-Arylhydrazone Derivatives of Megazol against *Trypanosoma cruzi*. Antimicrob Agents Chemother. 2010;54: 2023–31. doi:10.1128/AAC.01241-09

68. Sánchez-Moreno M, Gómez-Contreras F, Navarro P, Marín C, Olmo F, Yunta M, et al. Phthalazine derivatives containing imidazole rings behave as Fe-SOD inhibitors and show remarkable anti-T. cruzi activity in immunodeficient-mouse mode of infection. J Med Chem. 2012;55: 9900–13. doi:10.1021/jm3011004

69. Sánchez-Moreno M, Marín C, Navarro P, Lamarque L, García-España E, Miranda C, et al. *In vitro* and *in vivo* trypanosomicidal activity of pyrazole-containing macrocyclic and macrobicyclic polyamines: their action on acute and chronic phases of Chagas disease. J Med Chem. 2012;55: 4231–43. doi:10.1021/jm2017144

70. Sánchez-Moreno M, Sanz A, Gómez-Contreras F, Navarro P, Marín C, Ramírez-Macias I, et al. *In vivo* Trypanosomicidal Activity of Imidazole- or Pyrazole-Based Benzo[ g ]phthalazine Derivatives against Acute and Chronic Phases of Chagas Disease. J Med Chem. 2011;54: 970–9. doi:10.1021/jm101198k

71. Santos C, Loria R, Oliveira L, Kuehn C, Toldo M, Albuquerque S, et al. Effects of dehydroepiandrosterone-sulfate (DHEA-S) and benznidazole treatments during acute infection of two different *Trypanosoma cruzi* strains. Immunobiology. 2010;215: 980–6. doi:10.1016/j.imbio.2009.11.002

72. Saraiva J, Lira A, Esperandim V, da Silva Ferreira D, Ferraudo A, Bastos J, et al. (-)-Hinokinin-loaded poly(d,l-lactide-co-glycolide) microparticles for Chagas disease. Parasitol Res. 2010;106: 703–8. doi:10.1007/s00436-010-1725-1

73. Silva C, Batista DG, Batista M, Lionel J, Hammer E, Brun R, et al. *In vitro* and *in vivo* activity of the chloroaryl-substituted imidazole viniconazole against *Trypanosoma cruzi*. Parasitology. 2014;141: 367–73. doi:10.1017/S0031182013001601

74. Silva J, Guedes P, Zottis A, Balliano T, Nascimento Silva F, França Lopes L, et al. Novel ruthenium complexes as potential drugs for Chagas’s disease: enzyme inhibition and *in vitro*/*in vivo* trypanocidal activity. Br J Pharmacol. 2010;160: 260–9. doi:10.1111/j.1476-5381.2009.00524.x

75. Silva J, Pavanelli W, Pereira J, Silva J, Franco D. Experimental Chemotherapy against *Trypanosoma cruzi* Infection Using Ruthenium Nitric Oxide Donors. Antimicrob Agents Chemother. 2009;53: 4414–21. doi:10.1128/AAC.00104-09

76. Soeiro MN, de Souza E, da Silva C, Batista DG, Batista M, Pavão B, et al. *In vitro* and *in vivo* Studies of the Antiparasitic Activity of Sterol 14α-demethylase (CYP51) Inhibitor VNI against Drug-Resistant Strains of *Trypanosoma cruzi*. Antimicrob Agents Chemother. 2013;57: 4151–63. doi:10.1128/AAC.00070-13

77. Sülsen V, Frank F, Cazorla S, Anesini C, Malchiodi E, Freixa B, et al. Trypanocidal and Leishmanicidal Activities of Sesquiterpene Lactones from Ambrosia tenuifolia Sprengel (Asteraceae). Antimicrob Agents Chemother. 2008;52: 2415–9. doi:10.1128/AAC.01630-07

78. Sülsen V, Frank F, Cazorla S, Barrera P, Freixa B, Vila R, et al. Psilostachyin C: a natural compound with trypanocidal activity. Int J Antimicrob Agents. 2011;37: 536–43. doi:10.1016/j.ijantimicag.2011.02.003

79. Suryadevara P, Olepu S, Lockman J, Ohkanda J, Karimi M, Verlinde C, et al. Structurally Simple Inhibitors of Lanosterol 14α-Demethylase Are Efficacious In a Rodent Model of Acute Chagas Disease. J Med Chem. 2009;25: 3703–15. doi:10.1021/jm900030h

80. Valdez R, Tonin L, Ueda-Nakamura T, Silva S, Dias Filho B, Kaneshima E, et al. *In vitro* and *in vivo* trypanocidal synergistic activity of N-butyl-1-(4-dimethylamino)phenyl-1,2,3,4-tetrahydro-β-carboline-3-carboxamide associated with benznidazole. Antimicrob Agents Chemother. 2012;56: 507–12. doi:10.1128/AAC.05575-11

81. Villalta F, Dobish M, Nde P, Kleshchenko Y, Hargrove T, Johnson C, et al. VNI cures acute and chronic experimental Chagas Disease. J Infect Dis. 2013;208: 504–11. doi:10.1093/infdis/jit042

82. Waghabi M, de Souza E, de Oliveira G, Keramidas M, Feige J, Araújo-Jorge T, et al. Pharmacological Inhibition of Transforming Growth Factor Signaling Decreases Infection and Prevents Heart Damage in Acute Chagas’ Disease. Antimicrob Agents Chemother. 2009;53: 4694–701. doi:10.1128/AAC.00580-09

83. Zhu X, Liu Q, Yang S, Parman T, Green C, Mirsalis J, et al. Evaluation of arylimidamides DB1955 and DB1960 as candidates against visceral leishmaniasis and Chagas’ disease: in vivo efficacy, acute toxicity, pharmacokinetics, and toxicology studies. Antimicrob Agents Chemother. 2012;56: 3690–9. doi:10.1128/AAC.06404-11
